# Protective Effects of Selected Herbal Additives Against Ochratoxin A-Induced Toxicosis and Oxidative Damage in Rabbits

**DOI:** 10.3390/antiox15080954

**Published:** 2026-07-30

**Authors:** Kalina Zhivkova, Krassimira Gospodinova, Dimitrinka Zapryanova, Vesselin Ivanov, Galina Nikolova, Yanka Karamalakova, Stoycho Stoev

**Affiliations:** 1 Department of General and Clinical Pathology, Faculty of Veterinary Medicine, Trakia University, 6000 Stara Zagora, Bulgaria; kalina.zhivkova@trakia-uni.bg (K.Z.); krasimira.gospodinova@trakia-uni.bg (K.G.); 2 Department of Biochemistry, Faculty of Veterinary Medicine, Trakia University, 6000 Stara Zagora, Bulgaria; zaprianowa@abv.bg; 3 Department of Social Medicine, Health Management and Disaster Medicine, Faculty of Medicine, Trakia University, 6000 Stara Zagora, Bulgaria; veselin.ivanov@trakia-uni.bg; 4 Department of Medical Chemistry and Biochemistry, Medical Faculty, Trakia University, 6000 Stara Zagora, Bulgaria; galina.nikolova@trakia-uni.bg

**Keywords:** ochratoxin A, rabbits, herbal feed additives, oxidative stress, protection, antioxidant effect, silymarin, *Silybum marianum*, *Withania somnifera*, *Centella asiatica*

## Abstract

The protective effects of herbal feed additives *Withania somnifera*, *Silybum marianum*, *Centella asiatica*, and silymarin (administered at feed levels of 4000 ppm, 5000 ppm, 4600 ppm, and 25,000 ppm, respectively) against the toxic effects of ochratoxin A (OTA) (administered at 2 ppm) were investigated in 48 New Zealand White rabbits (37 days old) over an 80-day experimental period. A decrease in body weight was seen in rabbits exposed to OTA alone, but that decrease was less pronounced in rabbits receiving herbal supplements. The most severe lesions in OTA-treated rabbits were found in the liver, kidneys and spleen, while milder lesions were seen in the heart, intestine and lungs. The intensity of macroscopic, histopathological and biochemical changes was highest in rabbits exposed to OTA alone, followed by rabbits additionally supplemented with herbal additives, as evidenced by histopathological findings and serum levels of blood urea nitrogen (BUN), creatinine, aspartate aminotransferase (AST), and alanine aminotransferase (ALT). The most pronounced protective effects of the herbal additives were observed in the kidneys and liver. Oxidative stress was significantly increased in rabbits exposed to OTA alone, as evidenced by elevated reactive oxygen species (ROS), nitric oxide (●NO), ascorbyl radicals (●Asc), protein oxidation (PO), procollagen type I alpha 1 (COL1A1), malondialdehyde (MDA), kidney injury molecule-1 (KIM-1), hydroxyproline (Hyp), advanced glycation end products (AEGs), heme oxygenase-1 (HO-1), catalase (CAT), and 8-hydroxy-2′-deoxyguanosine (8-OHdG), as well decreased superoxide dismutase (SOD) and glutathione peroxidase-1 (GPx-1) activities, while protective effects were seen for all herbal additives, and better expressed for *Centella asiatica* and *Silybum marianum*.

## 1. Introduction

Mycotoxins are global foodborne contaminants posing a threat to animal/human health and are considered a major concern for food safety [1]. Mycotoxic nephropathy is a widespread kidney disease that primarily affects both animals and humans, and is mainly caused by ochratoxin A (OTA). OTA contamination of feed and food is widely documented [2], and spontaneous cases of mycotoxic nephropathy caused by this mycotoxin are commonly observed among pigs and poultry in Balkan countries [3], Europe, and Africa [4]. The toxicity of OTA has been attributed to multiple mechanisms, including inhibition of enzymes involved in phenylalanine metabolism, suppression of protein synthesis, increased lipid peroxidation, and impaired mitochondrial phosphate transport, leading to mitochondrial dysfunction and subsequent renal and hepatic injury, with the severity and target organs varying among animal species.

Rabbits are more sensitive to OTA than guinea pigs, rats, and mice [5]. OTA has been reported to exhibit teratogenic effects [6] and acute toxicity in young rabbits, with a reported LD50 of 10 mg OTA/kg body weight [7]. However, spontaneous cases of ochratoxicosis in rabbits have been rarely documented in European countries or elsewhere [8]. At the same time, OTA occurrence in rabbit feeds is reported worldwide. For example, 25% of rabbit feed samples in Argentina were found to be contaminated with OTA, while 78% of the *Aspergillus niger* var. *awamori* isolates recovered from rabbit feed were capable of producing OTA [9]. On the other hand, clinical, biochemical, and pathological features of OTA-toxicosis in rabbits [10,11,12,13], as well as possible preventive measures [14], remain insufficiently investigated. This circumstance highlights the need for more comprehensive studies on the characteristic symptoms of ochratoxicosis in rabbits with regard to diagnosis as well as possible preventive measures.

Plant-derived bioactive compounds have long been used in traditional human and veterinary medicine and are increasingly incorporated into animal nutrition as sustainable feed additives because of their antimicrobial, antioxidant, anti-inflammatory, and immunomodulatory properties [15,16]. Many drugs derived from plants have been developed based on knowledge of traditional methods of disease treatment provided by traditional healers [17]. In developing countries, a large proportion of the population still uses such traditional approaches for disease treatment, including medicinal plants or their extracts. Despite the extensive use of medicinal plants, fewer than 15% have been scientifically evaluated for their phytochemical content and biological activity [18]. Nowadays, herbal medicine is considered to be a safe alternative to synthetic drugs and, therefore, there is considerable interest in medicinal plants and their extracts. In this regard, further research, including animal studies, is essential to enhance our knowledge of the pharmacological effects and possible therapeutic uses of medicinal plants in both animal and human health.

The use of clay-based mycotoxin binders on a large industrial scale has certain limitations because they may also bind essential nutrients, thereby reducing the nutritional value of feed. In addition, the efficacy of many commonly used clay binders (e.g., bentonite, kaolin, and sepiolite) against ochratoxin A is often limited, highlighting the need for complementary protective strategies. It is well known that biological methods for preventing the toxic effects of OTA are safe and preserve the organoleptic and nutritional properties of feed. Therefore, this study was undertaken to evaluate the potential protective effects of selected herbal additives, namely *Centella asiatica* (Gotu kola), *Withania somnifera* (ashwagandha), and *Silybum marianum* (milk thistle), against OTA-induced alterations in renal, hepatic, and immune functions in rabbits. These herbs are also potent antioxidants and may ameliorate the deleterious effects of OTA, including oxidative stress and increased lipid peroxidation [19].

In addition to its protective effect against oxidative stress and oxidative damage, *Centella asiatica* (Gotu kola) is reported to ameliorate the harmful damages of free radicals on the gastrointestinal mucosa by improving the mucosal barrier. It exhibits anti-inflammatory activity in the gastrointestinal system and contributes to vascular integrity, potentially reducing OTA-induced intestinal mucosal injury and increased vascular permeability [19,20]. *Centella asiatica* has also been reported to improve growth performance and feed conversion efficiency in rabbits [21] as well as for its antibacterial and immunostimulatory properties [22,23], and could ameliorate OTA-induced immunosuppression and possible secondary infections [24,25,26]. This herb is also reported as a hepatoprotective and nephroprotective agent in humans [22] and chicks [23].

*Silybum marianum* (milk thistle) and its standardised seed extract silymarin used in the present study have also been reported to possess antioxidative, hepatoprotective and nephroprotective effects, in addition to immunostimulating, anti-lipoperoxidative, hypolipidemic, anti-inflammatory, and membrane-stabilising properties [27,28,29,30,31,32,33,34]. A hepatoprotective effect, as measured by a decrease in serum ALP (alkaline phosphatase), AST (aspartate aminotransferase) and ALT (alanine aminotransferase), and reduced lipid peroxidation, was reported in rats exposed to the hepatotoxic effect of NDEA (N-nitrosodiethylamine) when treated with silymarin [35]. Similar protective effects of *Silybum marianum* were also observed in chicks exposed to aflatoxins [34], in models of alcoholic liver damage in mice [31], and in diethylnitrosamine-induced liver damage in rats [29,30]. Moreover, *Silybum marianum* (whole dried plant) was found to improve the health performance of growing rabbits without adverse side effects on productive performance, carcass traits, or meat quality [36]. The oil from *Silybum marianum* was also found to significantly reduce serum cholesterol and triglyceride levels in rabbits [37]. *Silybum marianum* seed was also reported to attenuate morbidity and mortality in rabbits and to improve growth performance in HYLA broiler rabbits, suggesting a potential role in reducing the impact of disease in farming systems [38].

The herb *Withania somnifera* (ashwagandha) used in the present study has been reported to possess similar immunomodulating, anti-inflammatory, antioxidative, hypolipidemic, and hepatoprotective properties in laboratory animals [39,40,41] and chicks [23]. Moreover, *W. somnifera* was reported to reduce overall oxidative stress and improve health biomarkers in rabbits affected by hypercholesterolemia without adverse effects [42].

The purpose of this study was to evaluate the potential protective properties of *Silybum marianum*, *Centella asiatica*, *Withania somnifera*, and silymarin, administered as feed additives to rabbits [43], against the deleterious effects of OTA. Thus, these natural feed supplements were evaluated for the safe utilisation of rabbit feeds naturally contaminated with OTA, with the aim of preventing losses due to reduced weight gain in growing rabbits and the potential condemnation of contaminated feed.

The present study builds upon our previous findings in broiler chicks [23] by extending the evaluation of selected herbal feed additives to rabbits, a species known to be particularly sensitive to OTA. In addition, a broader panel of oxidative stress, inflammatory, and tissue injury biomarkers was included to provide a more comprehensive assessment of the protective effects of these additives, and the standardised milk thistle extract silymarin was evaluated as an additional protective agent.

## 2. Materials and Methods

### 2.1. OTA Production

The production of OTA was carried out by using *Aspergillus ochraceus* (isolate D2306) as previously described by Stoev et al. [25]. The material was then heat-treated at 80 °C for 1 h, and stored at −20 °C until use. HPLC analysis of a sample of this product confirmed the presence of approximately 2 mg/g OTA and a relatively small amount of the biologically inactive dechloro-analogue ochratoxin B. No other mycotoxins were produced in this fermentation process, and subsequent dilution (~10^3^-fold) when homogenised into the rabbit ration resulted in only a minimal addition of any other components. The ochratoxin-rich material was thoroughly homogenised into the rabbits’ feed to give the required contamination level of 2 ppm OTA in the diet. The final concentration of OTA was also verified by HPLC to confirm the target level of 2 ppm OTA. The commercially available standard complete feeds for growing rabbits were confirmed to be free of other mycotoxins.

### 2.2. Herbal Additives

Herbal supplements (*Centella asiatica*, *Withania somnifera*, *Silybum marianum*) were provided by Parceval Pharmaceuticals (Wellington 7654, South Africa) and consisted of root powder of *Withania somnifera radix* powder (400 μm particles), *Centella asiatica* leaf powder (400 μm particles), *Silybum marianum* fruit powder (400 μm particles), supplied in vacuum packs. Silymarin (milk thistle extract powder) was manufactured by Wuxi Gorunjie Technology Co., Ltd. (Wuxi, China). The doses of the herbs were calculated in advance, taking into account several factors, including body weight, dose conversion factors reported in the literature for rabbits, available WHO guidelines, and standard toxicological recommendations, to approximate effective doses against OTA toxicity. Data from previous experiments were also considered [19,23,34,41,44]. Additional adjustments were performed according to the breed and age of the rabbits. Preliminary investigations assessing the safety of the dietary concentrations of the herbal additives used in the present study demonstrated no adverse effects on the evaluated biochemical and oxidative stress parameters, nor any pathological alterations, in comparison with untreated control animals in both broiler chicks and rabbits.

### 2.3. Experimental Design

For the experimental setup, 48 New Zealand White rabbits aged 30 days and with a body weight of 0.600–0.700 kg were used. The rabbits were purchased immediately after weaning from “NIGO” Agricultural Institute Stara Zagora and housed in the Livestock Facility of the Agricultural Faculty of Trakia University. The rabbits were maintained under standardised conditions for this species, including natural temperature, controlled humidity, and a normal light regime, with free access to food and water. Housing conditions met the minimum requirements of 3500 cm^2^ floor area per rabbit and a minimum height of 45 cm. During a one-week adaptation period, rabbits were fed a standard complete diet for rabbits (free from OTA) containing the necessary vitamins and minerals (manufactured and purchased from Grainstore feeds AD, Sofia, Bulgaria) with drinking water available “ad libitum”. Thus, at the start of the 80-day experimental exposure period, rabbits were 37 days old. The animals were randomly assigned to five experimental groups and one control group, each consisting of eight animals (four males and four females), and there was no exclusion of animals.

Feed was prepared in advance by mixing OTA and herbal supplements into the standard complete diet (non-pelleted) at the following concentrations (Table 1):

✓ Group SIL + OTA: 2 ppm OTA + 25,000 ppm silymarin (2.5% of diet);
✓ Group CA + OTA: 2 ppm OTA + 4600 ppm *Centella asiatica*;
✓ Group WS + OTA: 2 ppm OTA + 4000 ppm *Withania somnifera*;
✓ Group SM + OTA: 2 ppm OTA + 5000 ppm *Silybum marianum*;
✓ Group OTA: 2 ppm OTA-only;
✓ Control group: no OTA or herbal additives.

After stepwise mixing and homogenization of the toxin and herbal supplements in the feed designed for each group, the same feed was granulated using an electric feed granulator GF-0893 (Golden Fish SRL, Ștefăneștii de Jos, Romania). Thorough disinfection was performed after each batch to prevent cross-contamination between groups. Feed and water were provided *ad libitum* and replenished daily throughout the 80-day experimental period.

During the experiment, prophylactic treatments were administered according to standard protocols for growing rabbits, including Egg booster (1 g/L drinking water from day 1 to day 4 and from day 14 to day 20), EsB3 (1 g/L—4 days during the adaptation period), and Baytril (5 mg/kg i.m. from day 14 to day 19), according to age and veterinary recommendations.

### 2.4. Measurements

Body weight was recorded on day 1 of the experiment (0.800–0.900 kg), after which animals were randomly assigned to groups. Body weight and feed intake were measured regularly throughout the experiment and at its conclusion.

### 2.5. Histological Examination

On day 30, four rabbits (two males and two females) from each group were euthanized using an overdose of anaesthetic (isoflurane inhalation), following prior sedation with Zoletil (intramuscular). The remaining animals were euthanized on day 80 using the same protocol. Following euthanasia, tissue samples were collected from internal organs for histological analysis, including liver, heart, kidneys, lungs, spleen, intestines, brain, cerebellum, thymus, lymph nodes, testes, and ovaries. Samples were fixed in 10% neutral buffered formalin and embedded in paraffin and stained with hematoxylin–eosin. Periodic acid–Schiff (PAS) stain was used to prove glycoproteins, lipoproteins, or mucoproteins in various tissues. Additional staining with Weigert’s iron hematoxylin was performed to assess fibrin deposition. Some samples were processed as frozen sections and stained with Sudan III to detect lipid accumulation.

### 2.6. Clinical Biochemistry

Blood for clinical biochemistry (2 mL) was collected on day 30 of the experiment. Blood samples (2 mL) were collected from either the auricular (*v. auricularis*) or jugular (*v. jugularis*) vein of each rabbit after sedation with acepromazine (0.75 mg/kg, i.m.). Within 1 h of collection, the samples were centrifuged, and 400 μL of serum was separated for clinical biochemical analyses.

The serum enzyme activities of aspartate aminotransferase (AST), alanine aminotransferase (ALT), alkaline phosphatase (ALP), as well as serum glucose (GLU) levels, were measured using commercial kits (Chema Diagnostica Srl, Monsano, Italy) on a Mindray BA-88 biochemical analyser (Shenzhen Mindray Bio-Medical Electronics Co., Ltd., Shenzhen, China). The serum levels of total protein (TP), albumin (ALB), cholesterol (CHOL), triglycerides (TGs), creatinine (CR) and blood urea nitrogen (BUN) were determined using Giesse Diagnostics kits (GIESSE DIAGNOSTICS SRL, Guidonia Montecelio, Italy).

### 2.7. Oxidant and Antioxidant Status Measurements

Materials for the assessment of oxidant and antioxidant status were collected from the liver, kidneys, pancreas and blood on day 30 of the experiment, as described above.

Antioxidant enzyme activities and biomarker levels, including CAT (U/mL), SOD (U/mL), GPx-1 (U/mL), HO-1 (pg/mL), MDA (µmol/mg), COL1A1 (pg/mL), SIRT-1 (pg/mL), 8-OHdG (ng/mL), AEGs (µg/mL), PC (nmol/mg), KIM-1 (ng/mL), TNF-α (pg/mL), IL-6 (pg/mL), IL-10 (pg/mL), IL-1β (ng/mL), and IFN-γ (pg/mL) were measured using ELISA kits (Cell Biolabs, San Diego, CA, USA).

Hydroxyproline (Hyp; μg/g tissue) was determined spectrophotometrically at 550 nm [45].

Oxidant status and radical formation, including ●NO (a.u.), ●Asc (a.u.) radicals, ROS production (a.u.), and PO (a.u.), were evaluated by EPR (Electron paramagnetic resonance) spectroscopy, and results were presented in arbitrary units (a.u). ROS production was assessed using N-tert-butyl-alpha-phenylnitrone (PBN) serving as a spin-trapping agent, following a modified methodology [46]. The ●NO generation, spin–adduct formed between the spin-trap carboxy 2-(4-carboxyphenyl)-4,4,5,5-tetramethyl (CPTIO.K), and ●NO radicals in systems were measured according to Georgiev et al. [47]. The ●Asc radicals were studied according to Bailey [48], using tissue/blood mixtures in DMSO (1:3 ratio). Protein oxidation (−SH group modifications) was assessed by conjugation with the spin-trap 3-maleimido proxyl (5-MSL; 1:2 ratio) [49].

The EPR analyses were conducted using five repeated measurements per sample, with the following parameters: 3503–3515 G centre field; 6.42–20.00 mW microwave power; 5–10 G modulation amplitude; and 1–5 scans per sample.

### 2.8. Statistical Methods

EPR measurements were performed with a Bruker BioSpin GmbH (Ettlingen, Germany). Spectral processing was carried out using Bruker WIN-EPR SimFonia 1.2/6130860 software after double integration, and the results were presented in arbitrary units (a.u.).

The clinical data were statistically processed using Statistica 8 (StatSoft, Inc., Tulsa, OK, USA). One-way ANOVA followed by Tukey’s post hoc test was used to assess significant differences between mean values of the investigated parameters among the different groups of rabbits. Statistical significance was set at *p* < 0.05.

## 3. Results

### 3.1. Clinical Observation

Reduced feed intake, lethargy, depression, and anorexia were observed only in a subset of rabbits exposed to feed contaminated with 2 ppm OTA without additional herbal supplementation, primarily during the final weeks of the experiment.

A few rabbits from the experimental groups showed respiratory distress and nasal discharge (snuffles), which disappeared after prophylactic treatment of all animals with Baytril at the beginning of the third week of the experiment.

### 3.2. Body Weight, Feed Intake and Feed Conversion Ratio (FCR)

Body weight was significantly reduced in rabbits fed OTA-contaminated feed, but the decrease was less pronounced in animals supplemented with herbal additives. Rabbits supplemented with *Centella asiatica* showed no significant difference in body weight compared with the control group, and a significant increase in body weight was observed in rabbits supplemented with *Centella asiatica* and *Withania somnifera* compared with the OTA group. Feed intake was reduced in all rabbits exposed to the OTA-contaminated diet, but this decrease was less pronounced in animals receiving additional herbal supplementation (Table 2). Regarding FCR, no significant differences were observed between experimental and control groups when feed consumption per kg body weight was compared, despite the fact that FCR was highest in rabbits exposed only to OTA and lowest in control rabbits.

In rabbits supplemented with herbal additives, FCR values were lower than in OTA-only rabbits but higher than in controls. The FCR of rabbits in the *Centella asiatica* group was closest to that of the control group.

### 3.3. Biochemical Changes

Serum glucose levels were decreased in response to OTA exposure in all experimental groups, and no protective effect was observed for any of the tested feed supplements. Serum albumin and total protein levels were slightly, but not significantly, decreased in the OTA-exposed group and in the group additionally supplemented with silymarin. Serum cholesterol levels were slightly, but not significantly, increased in all OTA-exposed groups, with the highest values observed in rabbits receiving OTA without herbal supplementation. Serum creatinine levels were increased in all OTA-exposed groups, except in the group supplemented with *Centella asiatica*, which appeared to prevent this increase. An increase in serum urea was observed in the OTA group and in groups supplemented with silymarin and *Centella asiatica*, whereas no such increase was detected in groups supplemented with *Silybum marianum* and *Withania somnifera*, suggesting a protective effect of these additives (Table 3). Serum triglyceride levels were elevated in response to OTA toxicity; however, significant protection against this increase was observed in rabbits supplemented with *Silybum marianum* and *Centella asiatica* (Table 4).

Serum AST activity was increased only in rabbits exposed to OTA without herbal supplementation and in those supplemented with silymarin; however, all tested herbal additives, including silymarin, showed a significant protective effect against this increase. A similar increase was observed in serum ALT and ALP activities in OTA-exposed rabbits; however, significant protection against an increase in ALP was mainly observed in the group supplemented with *Silybum marianum* (Table 4).

### 3.4. Gross Pathology

**Figure 1:**
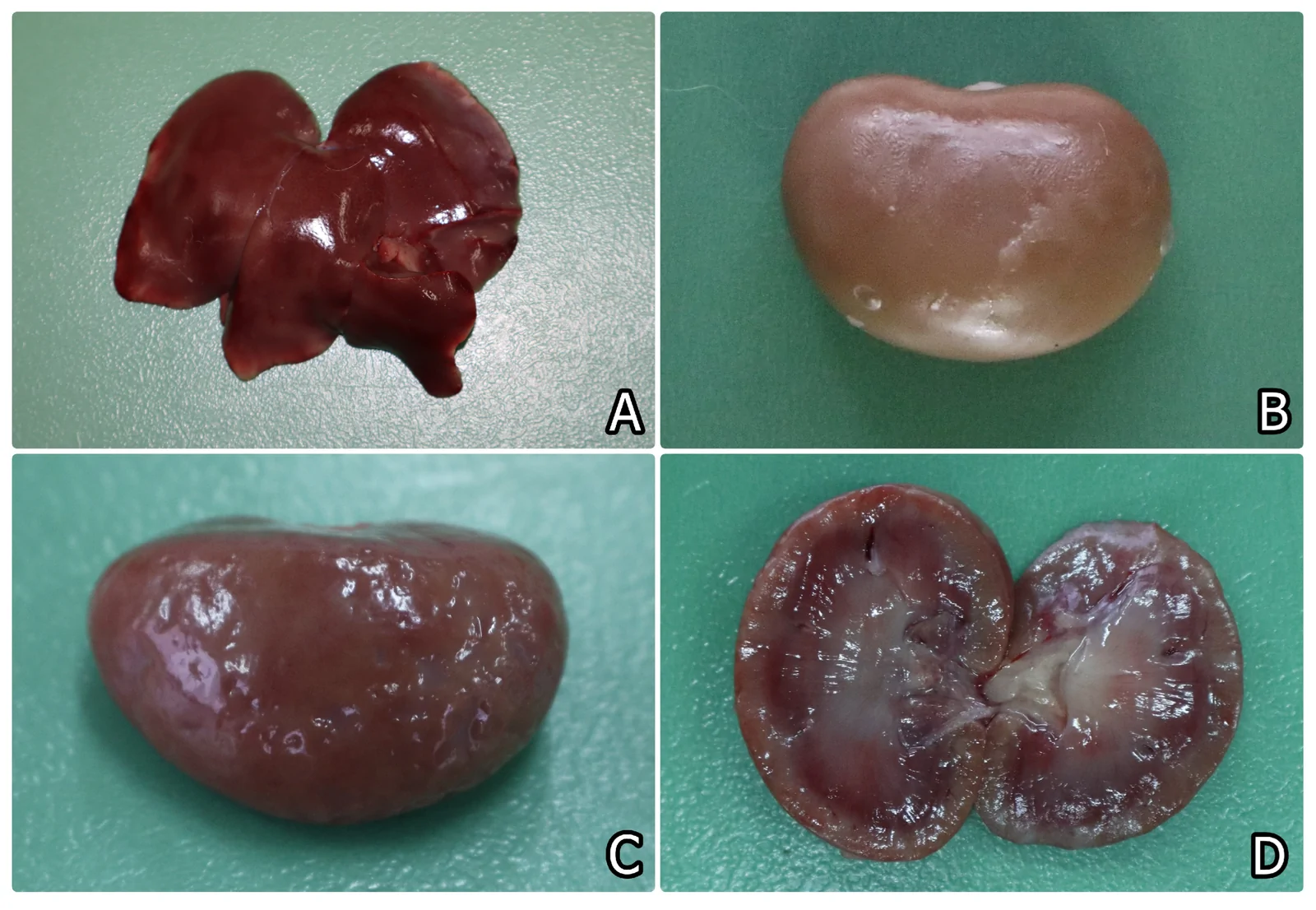
(**A**) Mottled surface of a liver from a rabbit in the OTA group on day 30 of the experiment; (**B**) pale cortex of a kidney from a rabbit in the OTA group on day 30 of the experiment; (**C**) furrowed surface of a kidney from a rabbit in the OTA group on day 80 of the experiment; (**D**) fibrotic changes on the cut surface of a kidney from a rabbit in the OTA group on day 80 of the experiment.

Macroscopic examination at necropsy revealed mild emaciation, primarily in rabbits exposed to OTA without herbal supplementation. Mild hyperaemia of the intestinal mucosa was observed, being more pronounced in rabbits exposed to OTA alone. The liver in these rabbits exhibited a mottled appearance, characterised by pale grey-brown areas on the surface (Figure 1A). The kidneys of rabbits in the OTA-only group showed a pale or mottled cortex (Figure 1B), clearly visible on the cut surface. Slight fibrotic changes and a furrowed renal surface were observed only in rabbits euthanized on day 80 of the experiment (Figure 1C,D). The intestinal contents of these rabbits contained small amounts of mucus. Lesions in the nasal cavity and trachea, including hyperaemia, erosions, and the presence of purulent yellow-green exudate on the mucosa, were observed in a few rabbits from the OTA group, most prominently on day 80 of the experiment. Similar, but less pronounced, macroscopic changes in the liver, kidneys, and lungs were also observed in a few rabbits from the groups supplemented with herbal additives.

### 3.5. Histopathology

Histopathological examination of various internal organs on days 30 and 80 revealed that degenerative changes were most pronounced in rabbits exposed to OTA alone, and less severe in rabbits supplemented with herbal additives (Table 5).

**Figure 2:**
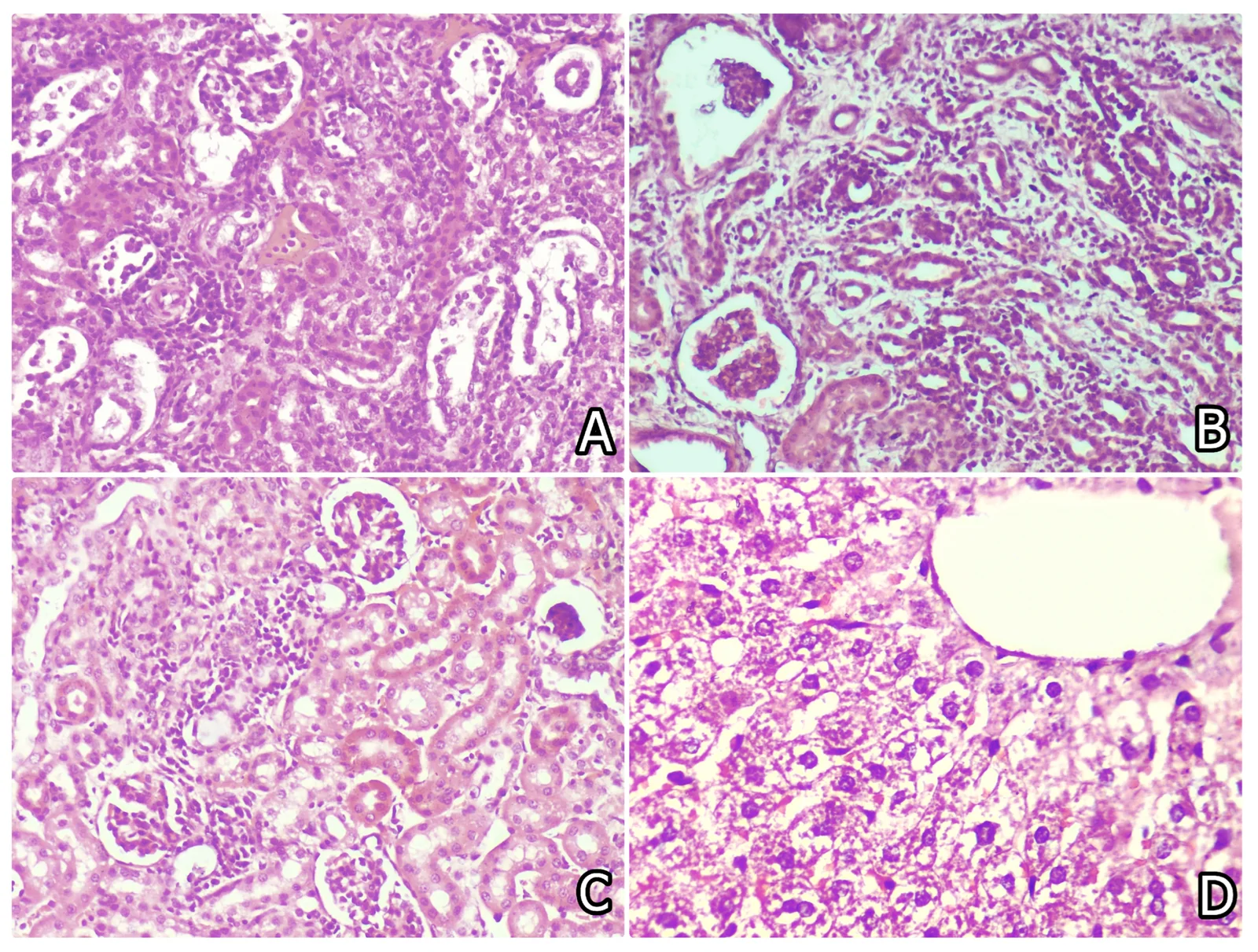
(**A**) Granular degeneration in proximal tubules, mononuclear infiltration in the interstitium, and initial atrophy of some tubules of kidneys from a rabbit in the OTA group on day 30 of the experiment. Kidney. H/E × 240. (**B**) Mononuclear and connective tissue proliferation, sclerosis of some glomeruli, and atrophy of tubules of kidneys from a rabbit in the OTA group on day 80 of the experiment. Kidney. H/E × 240. (**C**) Mild mononuclear proliferation in the interstitium, atrophy of a few tubules, and sclerosis of single glomeruli of kidneys from a rabbit in the WS+OTA group on day 30 of the experiment. Kidney. H/E × 240. (**D**) Vacuolar and granular degeneration of hepatocytes in the liver of a rabbit from the OTA group on day 30 of the experiment. Liver. H/E × 300.

Examination revealed marked degenerative changes in the epithelium of proximal renal tubules, including desquamation and disintegration of epithelial cells and the presence of cellular or hyaline casts in the tubular lumen, accompanied by activation of capillary endothelium, mononuclear infiltration in the interstitium (Figure 2A), mild hyperaemia of vessels and peritubular capillaries, and slight mesangial and/or endothelial proliferation in glomeruli (hypercellularity). These degenerative and proliferative changes, together with interstitial fibrosis, glomerular sclerosis, and tubular atrophy, were more pronounced on day 80 in rabbits exposed to OTA alone (Figure 2B) and were less severe or absent in groups receiving herbal supplementation. Mild interstitial fibrosis and tubular atrophy were observed mainly in rabbits supplemented with *Withania somnifera*, but were rarely detected in those receiving *Centella asiatica*, *Silybum marianum*, or silymarin on day 80. Focal glomerular sclerosis was observed in rabbits supplemented with *Withania somnifera*, *Centella asiatica*, and *Silybum marianum* (Figure 2C), whereas occasional focal proliferation of connective tissue was noted in rabbits receiving silymarin at that time (Table 5).

**Figure 3:**
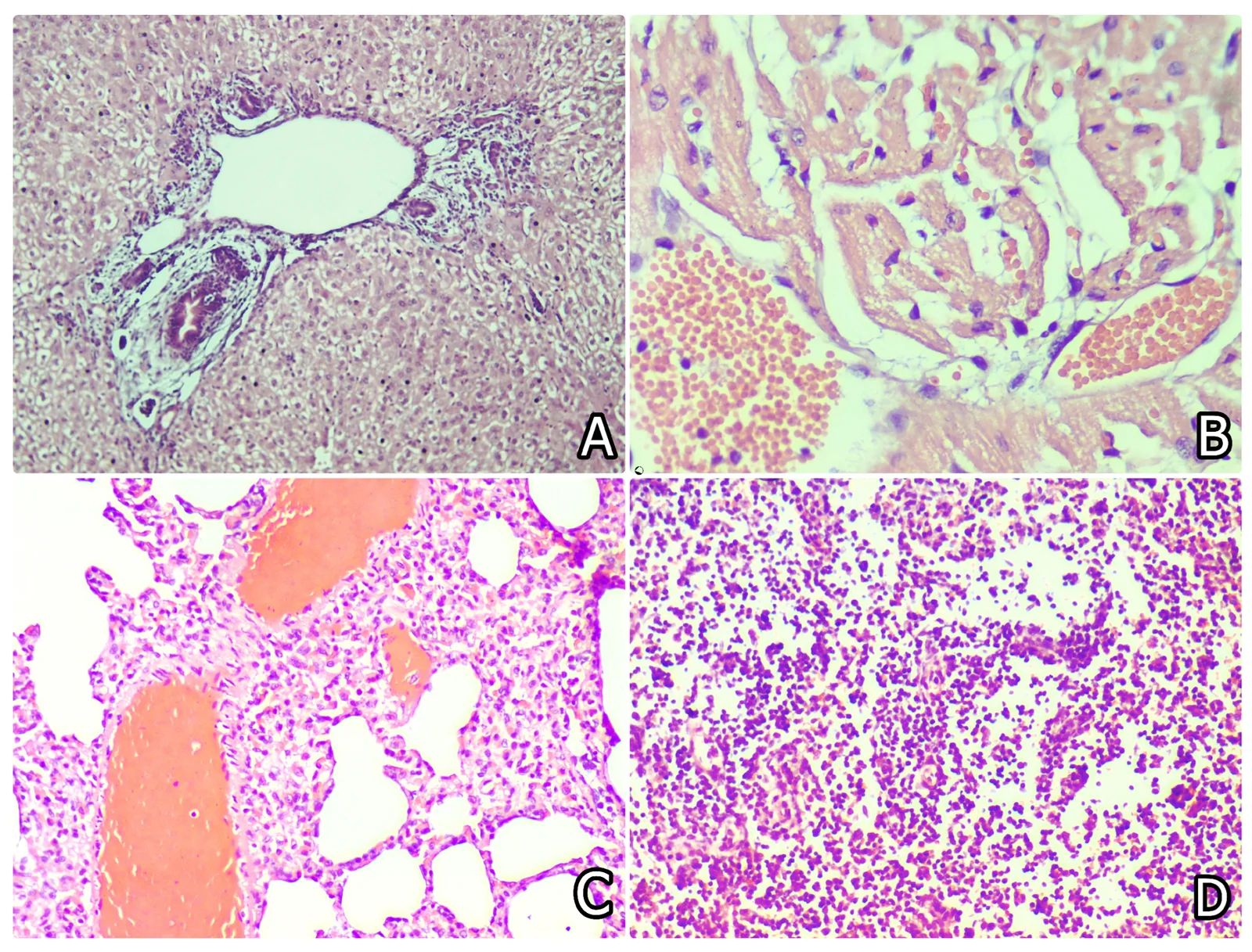
(**A**) Bile duct proliferation, peribiliary fibrosis, and oedema in the liver of a rabbit from the OTA group on day 80 of the experiment. Liver. H/E × 200. (**B**) Hyperaemia of vessels and lytic changes or vacuolation of myofibrils in the heart of a rabbit from the OTA group on day 30 of the experiment. Heart. H/E × 200. (**C**) Hyperaemia of vessels, focal pneumonic changes, and mononuclear infiltration in the interstitium of the lung of a rabbit from the OTA group on day 30 of the experiment. Lung. H/E × 200. (**D**) Degenerative changes and depletion of cells in the white pulp in the spleen of a rabbit from the OTA group on day 30 of the experiment. Spleen. H/E × 170.

In the liver, granular and/or vacuolar degeneration of hepatocytes (with karyopyknosis and karyolysis) was observed (Figure 2D), together with activation of Kupffer cells and/or sinusoidal endothelium, mild hyperaemia, peribiliary fibrosis and oedema, bile duct proliferation and perivascular mononuclear infiltration (Figure 3A). These changes were more pronounced in rabbits exposed to OTA alone and less severe in supplemented groups (Table 5). On day 80, these hepatic lesions were more advanced in the OTA-only group, with marked bile duct and mononuclear cell proliferation.

In the heart, mild degenerative and/or lytic changes in myofibrils, increased eosinophilia, mild hyperaemia, and mononuclear infiltration were observed mainly in rabbits exposed to OTA without supplementation (Figure 3B). These changes were less pronounced in supplemented groups. Similar findings were observed on day 80 (Table 5).

In the lung, mild hyperaemia, activation of alveolar macrophages, presence of siderocytes, focal inflammatory lesions (including pneumonia and epithelial proliferation), and perivascular or peribronchial mononuclear infiltration (Figure 3C) were primarily observed in the OTA group and only rarely in supplemented groups. These lesions were similar or more pronounced on day 80 in the OTA group (Table 5).

**Figure 4:**
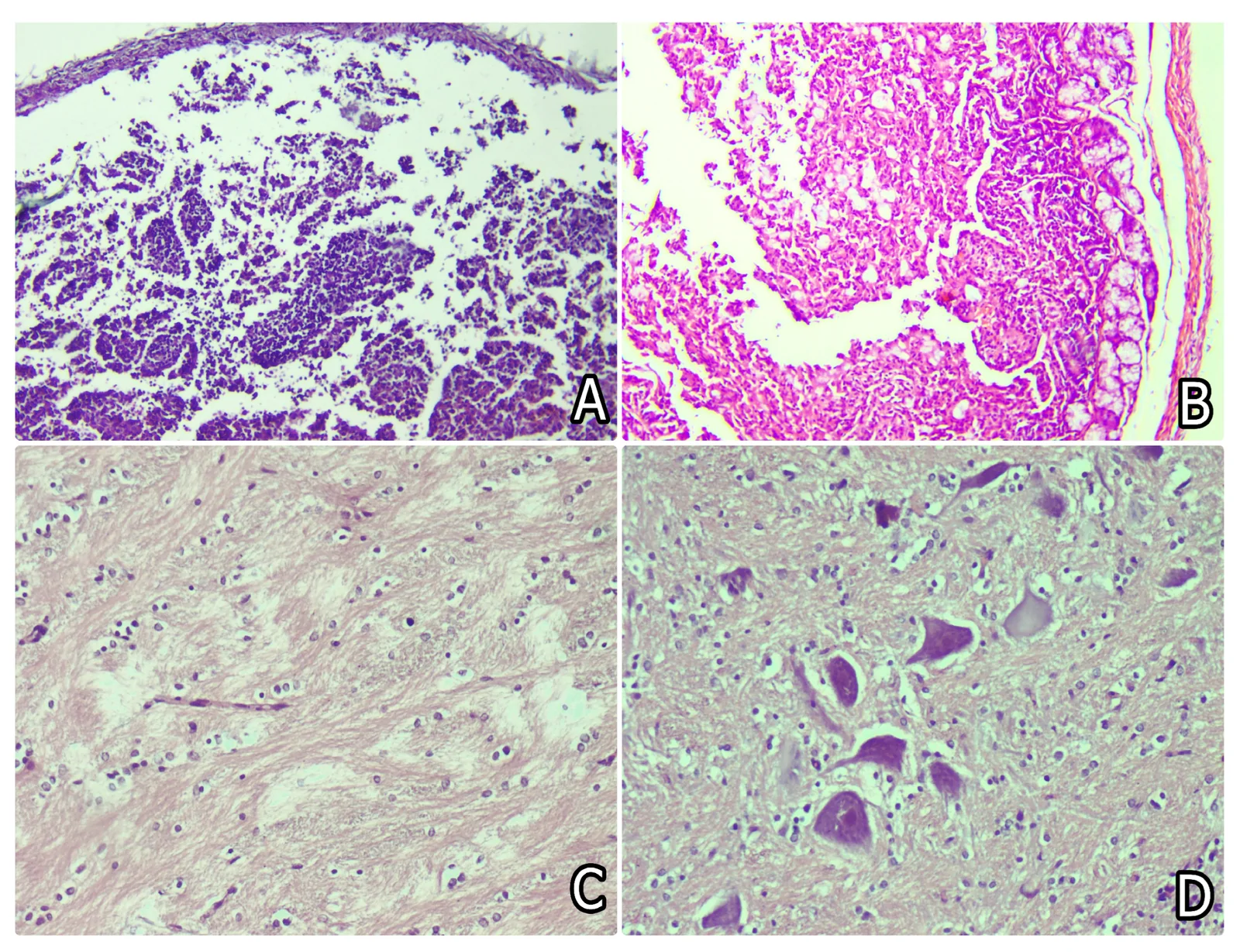
(**A**) Degenerative changes and depletion of cells in the white pulp in the spleen of a rabbit from the CA + OTA group on day 30 of the experiment. Spleen. H/E × 100. (**B**) Degenerative changes in superficial and glandular epithelium of mucosa of rabbit from OTA group on day 30 of experiment. Small intestine. H/E × 100. (**C**) Lytic changes in the brain substance of rabbit from OTA group on day 30 of experiment. Brain. H/E × 240. (**D**) Lytic or pyknotic changes in tigroid substance of neurons in brain of rabbit from OTA group on day 30 of experiment. Brain. H/E × 300.

In the spleen, a reduction in white pulp size and degenerative changes or depletion of cells were seen in lymphoid tissues (Figure 3D), with predominance of red pulp. Similar changes, including lymphoid depletion, were noted in the thymus cortex. These findings were most pronounced in the OTA-only group, followed by the *Centella asiatica* (Figure 4A) and *Silybum marianum* groups, and were minimal in rabbits receiving silymarin and *Withania somnifera*. Lesion severity was comparable on days 30 and 80 (Table 5).

In the intestine, degenerative changes in the superficial and glandular epithelium, along with mild hyperaemia, were mainly observed in OTA-exposed rabbits (Figure 4B) and were less pronounced in supplemented groups. No significant differences in the intensity of pathology were seen on days 30 and 80.

**Figure 5:**
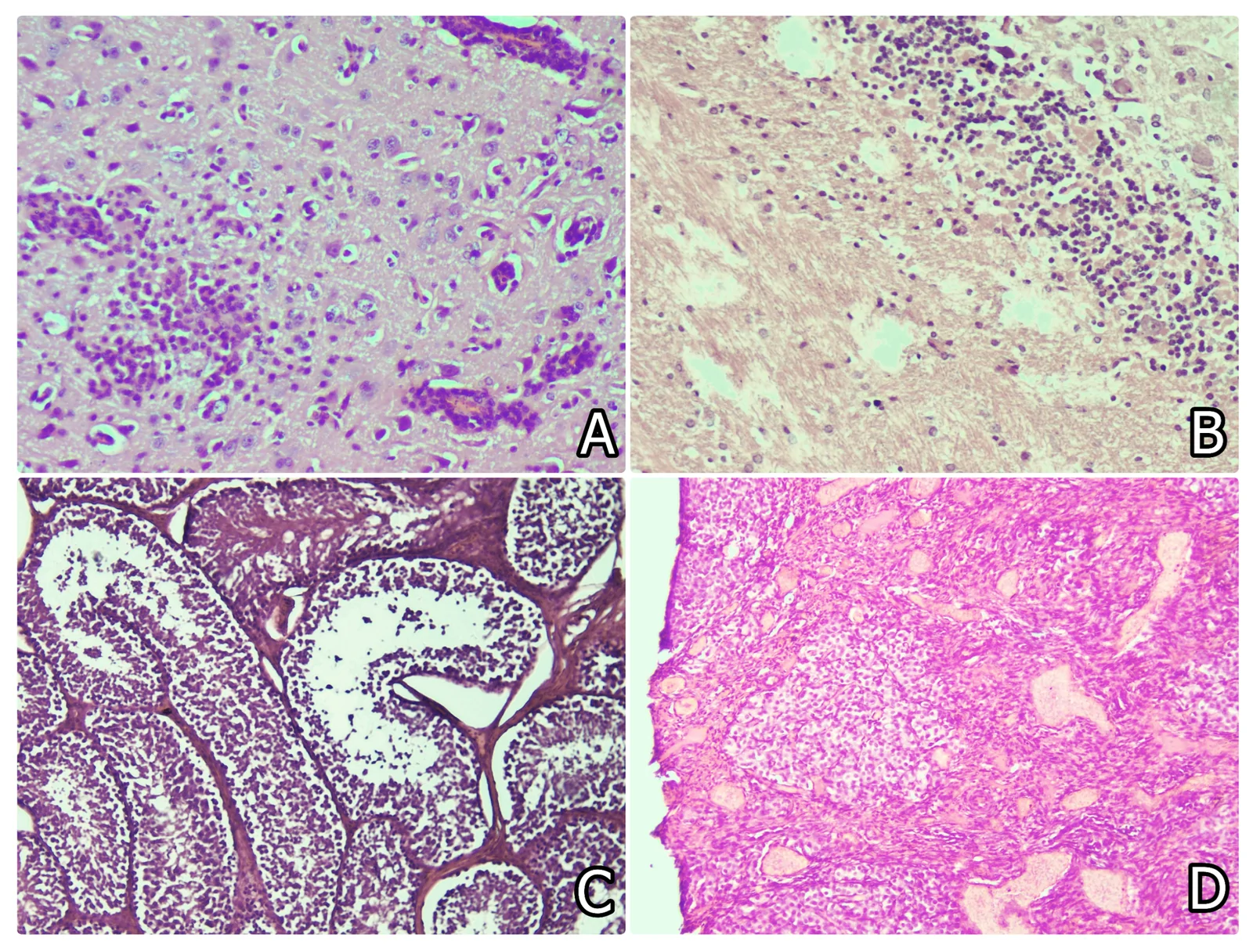
(**A**) Local proliferation of glia cells and pericapillary lymphocytic infiltration in the brain of a rabbit from the OTA group on day 30 of the experiment. Brain. H/E × 240. (**B**) Focal lytic changes in the region of *lamina medullaris* in the cerebellum of a rabbit from the OTA group on day 30 of the experiment. Cerebellum. H/E × 240. (**C**) Focal degenerative changes in seminiferous tubules in the testicle of a rabbit from the OTA group on day 80 of the experiment. Testicle. H/E × 200. (**D**) Proliferation of connective tissue and slight interstitial oedema in the ovary of a rabbit from the OTA group on day 30 of the experiment. Ovary. H/E × 200.

In the brain, mild lytic changes in brain tissue (Figure 4C), lytic or pyknotic changes in neurons (Figure 4D), and slight pericellular and/or pericapillary oedema were observed mainly in OTA-exposed rabbits and were less pronounced in supplemented groups. Focal microglial proliferation was observed around damaged neurons, along with lymphocytic infiltration around some vessels (Figure 5A). These changes were more pronounced on day 80, but only in the OTA group (Table 5).

In the cerebellum, mild oedema and lytic changes in the Purkinje cell layer and *lamina medullaris* were observed mainly in OTA-exposed rabbits (Figure 5B) with less pronounced changes in supplemented groups. No significant differences in the intensity of pathology were seen on days 30 and 80 (Table 5).

In the testicles, degenerative changes in seminiferous tubules (Figure 5C) and interstitial oedema were observed mainly in OTA-exposed rabbits on day 80 and were less pronounced in supplemented groups (Table 5).

In the ovary, mild hyperaemia, interstitial oedema, and connective tissue proliferation were observed mainly in OTA-exposed rabbits on days 30 (Figure 5D) and 80, with less pronounced changes in supplemented groups (Table 5).

No histopathological lesions were observed in the control group.

### 3.6. Oxidative Stress (OS) Investigations

**Figure 6:**
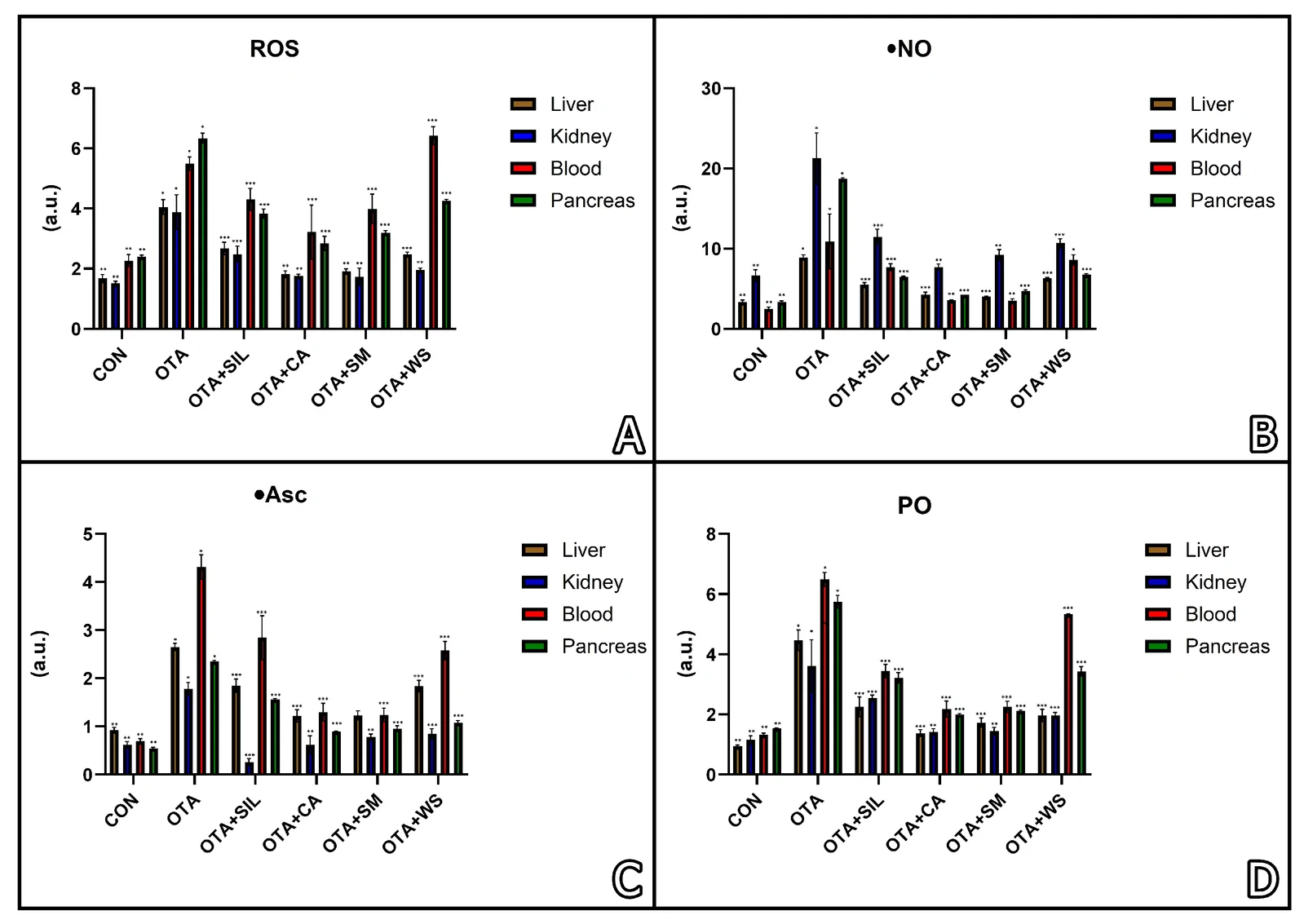
(**A**) ROS production in the liver, kidney, blood, and pancreas of rabbits supplemented with various putative antidotes and/or OTA for 30 days; (**B**) ●NO concentration in the liver, kidney, blood, and pancreas of rabbits supplemented with various putative antidotes and/or OTA for 30 days; (**C**) ●Asc radical levels in the liver, kidney, blood, and pancreas of rabbits supplemented with various putative antidotes and/or OTA for 30 days; (**D**) PO levels in the liver, kidney, blood, and pancreas of rabbits supplemented with various putative antidotes and/or OTA for 30 days. * Significant difference compared to the control group (*p* < 0.05). ** Significant difference compared to the OTA-group (*p* < 0.05). *** Significant difference compared to the control group and the OTA-group (*p* < 0.05).

The production of reactive oxygen species (ROS) (*p* < 0.05; Figure 6A), nitric oxide (●NO) (*p* < 0.05; Figure 6B), ascorbate radicals (●Asc) (*p* < 0.05; Figure 6C), and protein oxidation markers (PO; SH conformational/albumin changes) (Figure 6D), all indicative of oxidative stress (OS), was significantly increased in OTA-treated rabbits and reduced in rabbits supplemented with herbal additives compared with OTA-only animals. The strongest protective effects were observed in rabbits supplemented with *Centella asiatica* and *Silybum marianum*, followed by silymarin and *Withania somnifera*.

**Figure 7:**
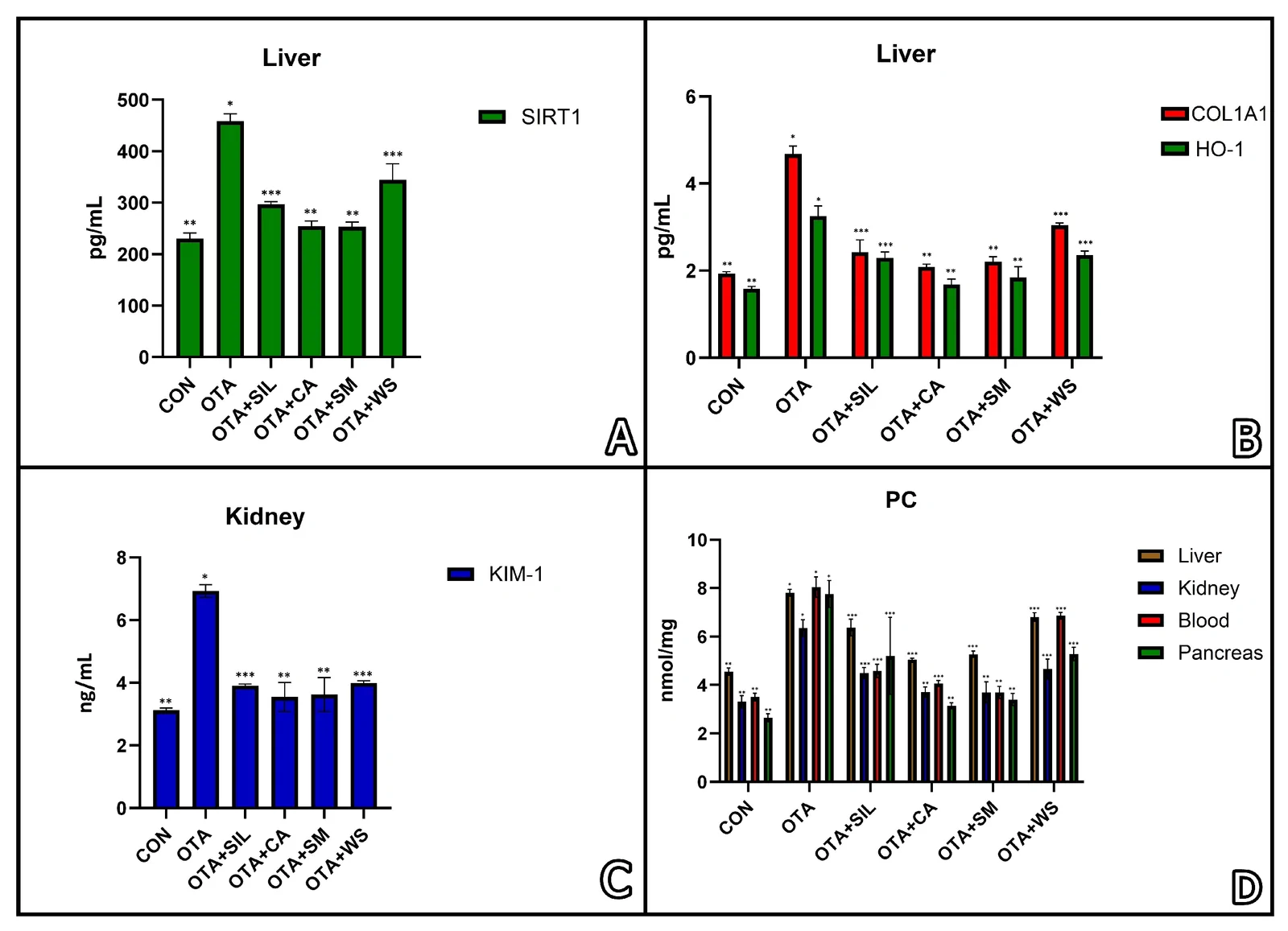
(**A**) SIRT-1 measured in the liver of rabbits supplemented with various putative antidotes and/or OTA for 30 days; (**B**) COL1A1/HO-1 measured in the liver of rabbits supplemented with various putative antidotes and/or OTA for 30 days; (**C**) KIM-1 measured in the kidney of rabbits supplemented with various putative antidotes and/or OTA for 30 days; (**D**) PC measured in the liver, kidney, blood and pancreas of rabbits supplemented with various putative antidotes and/or OTA for 30 days. * Significant difference compared to the control group (*p* < 0.05). ** Significant difference compared to the OTA-group (*p* < 0.05). *** Significant difference compared to the control group and the OTA-group (*p* < 0.05).

Oxidative stress biomarkers, including sirtuin 1 (SIRT-1) (*p* < 0.05) and procollagen I alpha 1 (COL1A1) (*p* < 0.05), indicated significantly increased liver damage in OTA-exposed rabbits, which was attenuated in groups receiving herbal supplementation (Figure 7A,B). Heme oxygenase-1 (HO-1) activity in the liver (*p* < 0.05; Figure 7B), kidney injury molecule-1 (KIM-1) levels in the kidney (*p* < 0.05; Figure 7C), and protein carbonyls (PCs) (*p* < 0.05; Figure 7D) were also significantly elevated in OTA-exposed rabbits and reduced in supplemented groups. *Centella asiatica* and *Silybum marianum* exhibited the strongest protective effects on both liver (Figure 7A,B) and kidney (Figure 7C) parameters.

**Figure 8:**
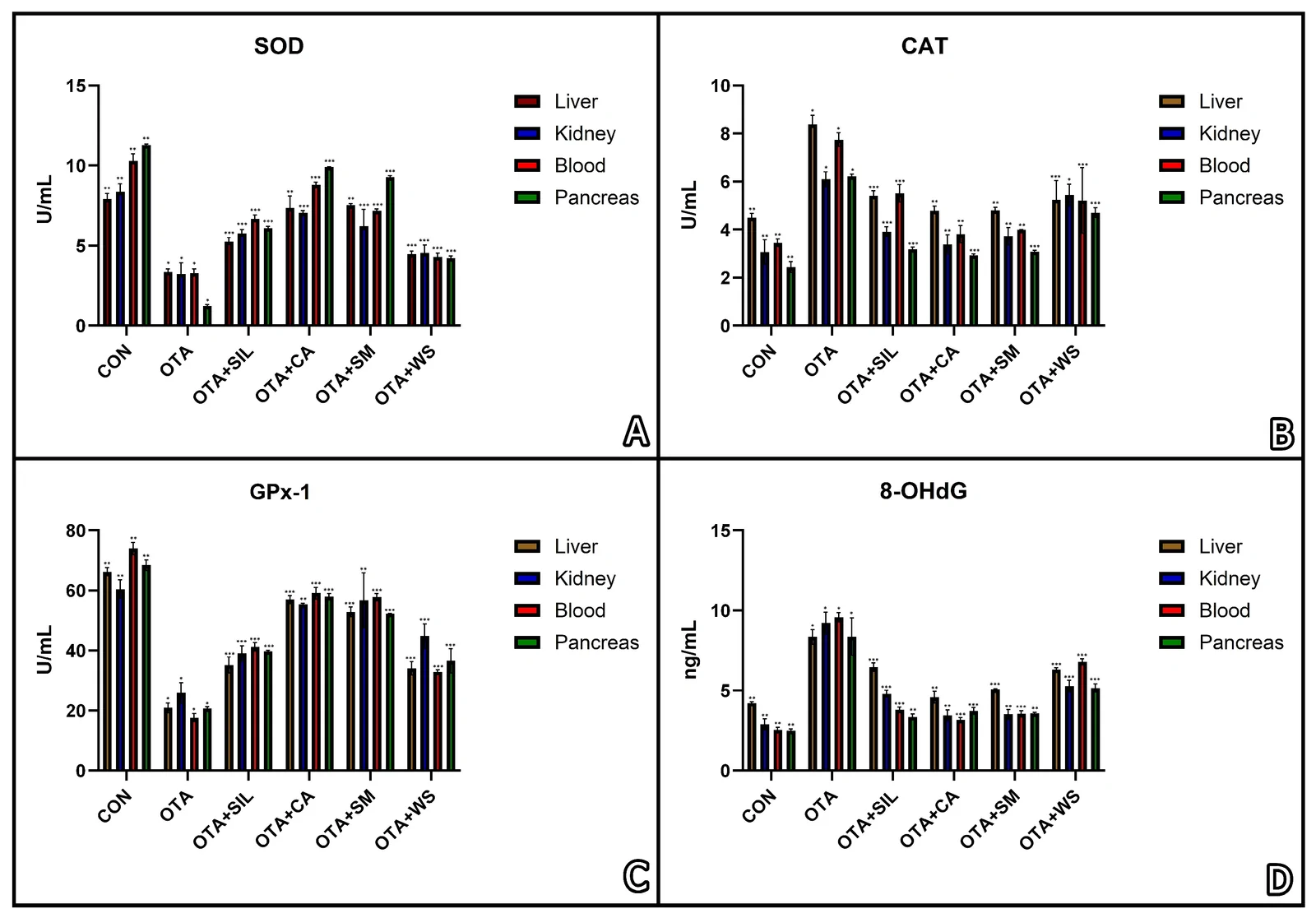
(**A**) SOD activity in the liver, kidney, blood and pancreas of rabbits supplemented with various putative antidotes and/or OTA for 30 days; (**B**) CAT activity in the liver, kidney, blood and pancreas of rabbits supplemented with various putative antidotes and/or OTA for 30 days; (**C**) GPx-1 activity in the liver, kidney, blood and pancreas of rabbits supplemented with various putative antidotes and/or OTA for 30 days; (**D**) 8-OHdG measured in the liver, kidney, blood and pancreas of rabbits supplemented with various putative antidotes and/or OTA for 30 days. * Significant difference compared to the control group (*p* < 0.05). ** Significant difference compared to the OTA-group (*p* < 0.05). *** Significant difference compared to the control group and the OTA-group (*p* < 0.05).

The activities of antioxidant enzymes superoxide dismutase (SOD) (*p* < 0.05; Figure 8A) and glutathione peroxidase-1 (GPx-1) (*p* < 0.05; Figure 8C) were significantly reduced in OTA-treated rabbits but significantly increased in animals receiving herbal supplementation. An opposite trend was observed for catalase (CAT) activity (*p* < 0.05; Figure 8B). Levels of 8-hydroxy-2′-deoxyguanosine (8-OHdG) (*p* < 0.05; Figure 8D), a marker of oxidative DNA damage, were increased in OTA-treated rabbits (liver, kidneys, blood, and pancreas) and reduced in supplemented groups.

**Figure 9:**
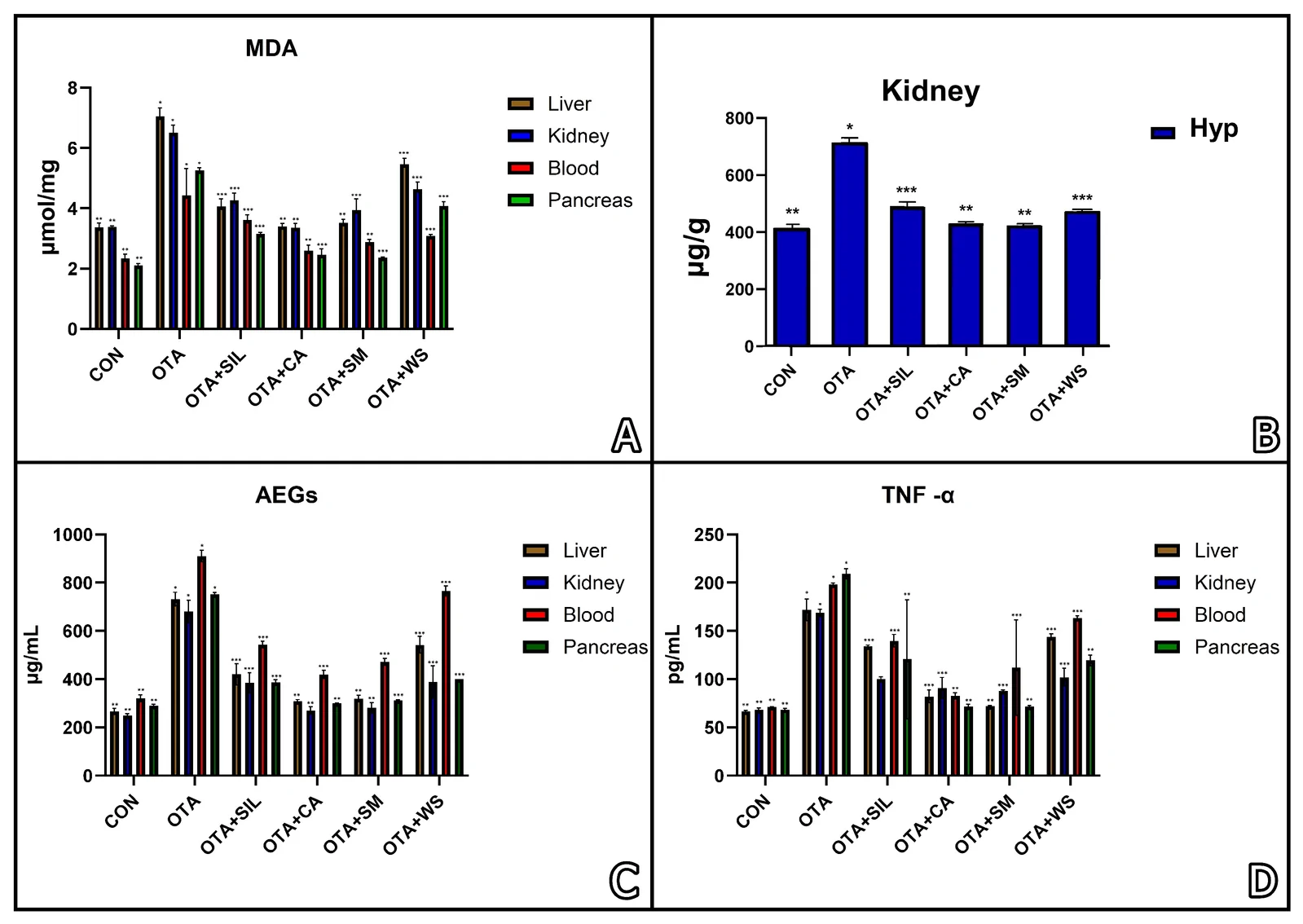
(**A**) Lipid peroxidation (MDA) measured in the liver, kidney, blood and pancreas of rabbits supplemented with various putative antidotes and/or OTA for 30 days; (**B**) Hyp concentration in the kidneys of rabbits supplemented with various putative antidotes and/or OTA for 30 days; (**C**) AEGs measured in the liver, kidney, blood and pancreas of rabbits supplemented with various putative antidotes and/or OTA for 30 days; (**D**) TNF-α measured in the liver, kidney, blood and pancreas of rabbits supplemented with various putative antidotes and/or OTA for 30 days. * Significant difference compared to the control group (*p* < 0.05). ** Significant difference compared to the OTA-group (*p* < 0.05). *** Significant difference compared to the control group and the OTA-group (*p* < 0.05).

Lipid peroxidation (a key indicator of oxidative lipid changes), assessed by malondialdehyde (MDA) levels (*p* < 0.05; Figure 9A), was increased in OTA-treated rabbits. Hydroxyproline (Hyp) level in the kidneys (Figure 9B) was increased in OTA-treated rabbits, but that increase was less pronounced in supplemented groups. Advanced glycation end products (AEGs) (Figure 9C) and tumour necrosis factor-α (TNF-α) (Figure 9D) were also increased in OTA-exposed rabbits but attenuated in supplemented groups.

**Figure 10:**
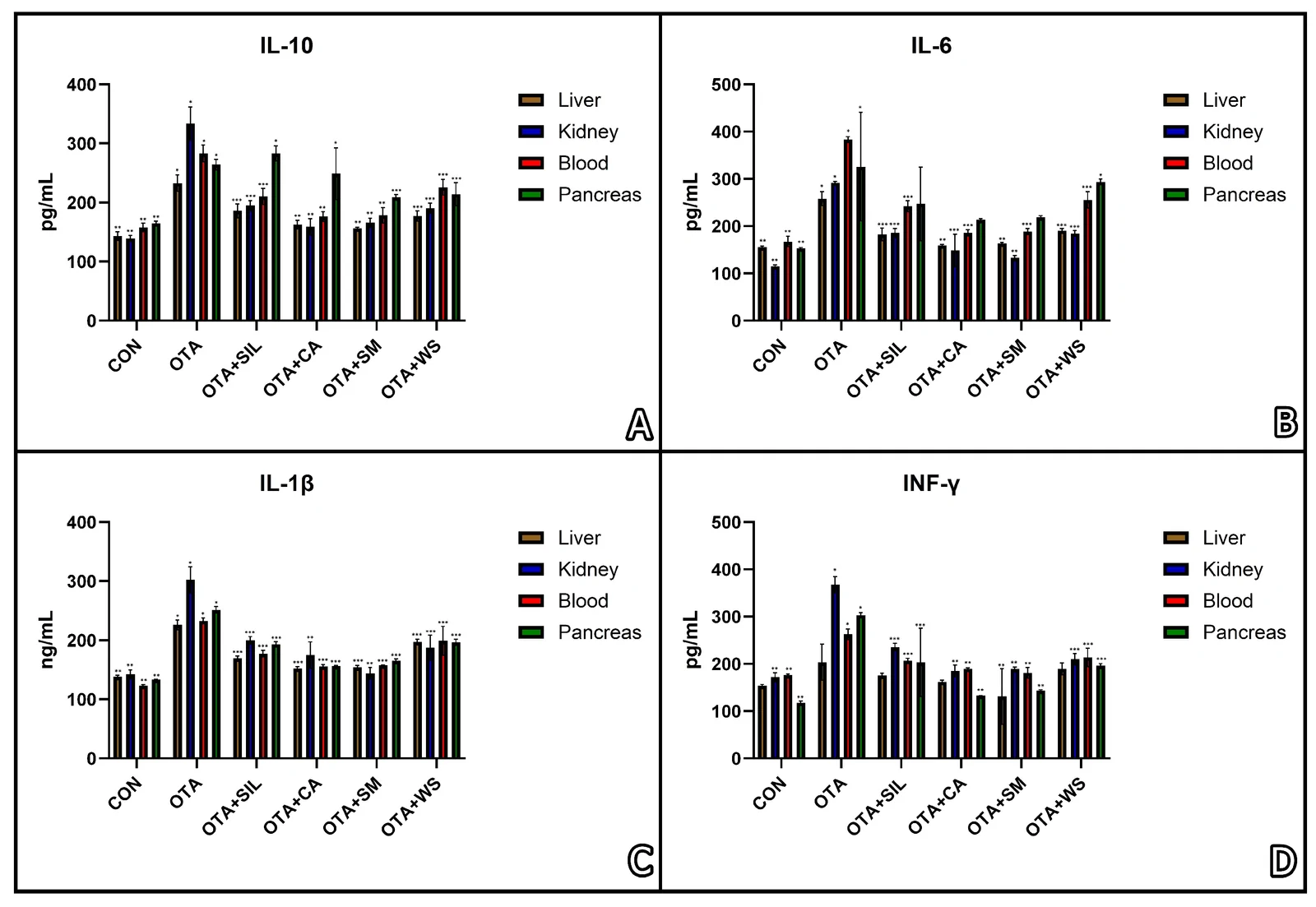
(**A**) IL-10 measured in the liver, kidney, blood, and pancreas of rabbits supplemented with various putative antidotes and/or OTA for 30 days; (**B**) IL-6 measured in the kidneys of rabbits supplemented with various putative antidotes and/or OTA for 30 days; (**C**) IL-1β measured in the liver, kidney, blood and pancreas of rabbits supplemented with various putative antidotes and/or OTA for 30 days; (**D**) INF-γ measured in the liver, kidney, blood, and pancreas of rabbits supplemented with various putative antidotes and/or OTA for 30 days. * Significant difference compared to the control group (*p* < 0.05). ** Significant difference compared to the OTA-group (*p* < 0.05). *** Significant difference compared to the control group and the OTA-group (*p* < 0.05).

Pro-inflammatory cytokines, including IL-10 (Figure 10A), IL-6 (Figure 10B), IL-1β (Figure 10C), and interferon-γ (IFN-γ) (Figure 10D), were elevated in the liver, kidneys, blood, and pancreas of OTA-exposed rabbits, with less pronounced increases in animals receiving herbal supplementation.

## 4. Discussion

Following the analysis of macroscopic and histopathological alterations, body weight reduction, and biochemical changes, it was evident that these effects were most pronounced in rabbits exposed to OTA without any herbal supplementation. These were followed by rabbits from the experimental groups receiving additional protection with various herbal additives. Protective effects against renal and hepatic damage were more clearly expressed in rabbits supplemented with *Centella asiatica*, *Withania somnifera*, or *Silybum marianum*, as indicated by both histopathological findings and biochemical parameters such as BUN, creatinine, AST, and ALP.

The elevation in BUN and serum creatinine levels in OTA-exposed rabbits [12,50] indicates impaired renal function and kidney damage [51,52]. This impairment may also explain the observed decrease in serum glucose and total protein levels, likely due to increased urinary excretion. Furthermore, OTA has been reported to induce glycogen accumulation in the liver by inhibiting glycogenolysis, resulting in decreased serum glucose levels [12]. The hepatic lesions observed in OTA-exposed rabbits may further contribute to disturbances in carbohydrate metabolism and the subsequent reduction in serum glucose [53,54]. Reduced serum glucose levels have also been associated with the development of neurological signs in rabbits dying from ochratoxicosis [53].

Additionally, decreased red blood cell (RBC) count and haemoglobin levels in OTA-intoxicated rabbits [55,56] may result in increased anaerobic metabolism and reduced oxygen supply to tissues, thereby contributing to degenerative changes in various organs.

The increase in serum triglycerides and cholesterol observed in OTA-exposed rabbits has been attributed to hepatocellular damage and disturbances in lipid metabolism, potentially associated with enhanced lipid peroxidation [57]. OTA-induced increase in lipid peroxidation [25,58] may lead to structural damage of cellular membranes, increased calcium influx, and subsequent cell necrosis [59].

The slight decrease in serum total protein and albumin levels may be associated with chronic liver damage induced by OTA, given the liver’s central role in plasma protein synthesis. Additionally, increased urinary protein loss due to renal damage may contribute to this effect [10,11]. Factors such as anorexia, reduced feed intake, and malabsorption resulting from OTA-induced gastrointestinal damage may further contribute to hypoproteinemia [12].

The increased serum activities of AST, ALT, and ALP in rabbits fed OTA-contaminated diets, as also reported by other authors [12,50], further support the presence of hepatic damage observed in the present study.

The reduction in body weight observed in OTA-treated animals may result from impaired protein synthesis induced by the mycotoxin [23,24,43,60,61,62], as well as from gastrointestinal damage leading to reduced nutrient absorption and poor digestion. Additionally, nutrient redistribution toward tissue repair and regeneration of damaged organs may contribute to reduced growth performance [63].

The gross pathological changes observed in the kidneys and liver (pale or mottled appearance), as well as the histopathological lesions affecting proximal tubular epithelium of the kidney and hepatocytes, are likely related to the primary routes of OTA excretion via the kidneys and liver, as well as its enterohepatic recirculation [64,65]. These processes may result in direct toxic effects on these organs. The membrane organic anion transport system has been suggested to facilitate OTA accumulation in proximal tubular epithelial cells [66]. The higher concentrations of OTA detected in kidneys, liver, and muscle tissue in rabbits fed diets containing 1–2 ppm OTA [10,11] may explain the pronounced pathological alterations observed in these organs in the present study, as well as in previous investigations [50,52]. Notably, OTA residues have been detected in these tissues up to four weeks after cessation of exposure, further contributing to sustained tissue damage [10,11].

The slight degenerative and/or lytic changes in cardiac myofibrils, together with mild mononuclear cell proliferation observed in rabbits exposed to OTA in the present study, have also been reported in New Zealand White male rabbits [50] and in newborn rabbits exposed to OTA via maternal transfer [52].

Degenerative alterations and depletion of lymphoid cells in the spleen and thymus of OTA-exposed rabbits [67] may be associated with impaired protein synthesis in lymphocytes, leading to reduced differentiation and proliferation, as suggested by previous studies [23,68,69]. This reduction in immunocompetent cells may result in suppression of both humoral and cellular immune responses.

The slight increase in body weight observed in rabbits fed diets supplemented with silymarin or *Silybum marianum* in the present study is consistent with findings in aflatoxin-exposed chicks [34,70]. Improvements in body weight gain, feed intake, and feed conversion ratio have been reported following supplementation with silymarin or *Silybum marianum*, with protective effects comparable to those of toxin binders [34]. Similar increases in body weight have also been observed in rats supplemented with *Withania somnifera* [39] or *Centella asiatica* [71]. These findings are in agreement with the present results, where a slight increase in body weight and improved feed utilisation were observed in rabbits receiving these herbal additives in addition to OTA exposure. Notably, both control rabbits and those supplemented with herbal additives exhibited better feed utilisation compared to rabbits exposed to OTA alone, with the most pronounced protective effect observed in rabbits supplemented with *Centella asiatica*.

A marked hepatoprotective and/or renoprotective effect of silymarin has been demonstrated against salinomycin-induced toxicity in adult rabbits [72], isoniazid-induced liver injury in rabbits [73], and nickel chloride-induced haematological and biochemical alterations in male rabbits [74]. Furthermore, protective effects of silymarin have been reported against cisplatin-induced renal damage and lipid peroxidation in rats [44], gentamicin-induced nephrotoxicity in dogs [75], and alloxan-induced diabetic nephropathy in rats [76].

Similarly, the hepatoprotective effects of *Silybum marianum* have been documented in experimental models of N-nitrosodiethylamine (NDEA)- and carbon tetrachloride (CCl_4_)-induced liver injury in rats [35], as well as in aflatoxin-exposed chicks [34,70]. These effects are evidenced by reductions in serum AST, ALT, and ALP activities, findings that are consistent with the results of the present study. These enzymes are well-established sensitive biomarkers of hepatic injury. The nephroprotective effects of *Silybum marianum* and silymarin observed in this study are supported by the lower serum levels of creatinine and BUN, as well as by the less severe histopathological lesions in the kidneys of treated rabbits.

The protective mechanisms of *Silybum marianum* and its seed extract silymarin are primarily attributed to their ability to reduce lipid peroxidation and enhance endogenous antioxidant defences, thereby preserving cellular membrane integrity [29,77]. These effects are largely mediated by flavonoid compounds such as silybin, isosilybin, silydianin, and silychristin [28], with silybin being the most biologically active component, exerting both protective and regenerative effects on the liver and kidneys in response to various toxic insults [28,78].

*Silybum marianum* has therefore been proposed as an effective feed additive for protecting poultry against the toxic effects of mycotoxins such as OTA and aflatoxins [23,34,70].

The herbal supplement *Withania somnifera* has been reported to exhibit pronounced hepatoprotective and antioxidant properties against carbon tetrachloride-induced liver injury in rats, as evidenced by changes in serum ALT, AST, ALP, and LDH (lactate dehydrogenase) levels, as well as histopathological findings [41]. These observations are consistent with the results of the present study. The modest protective effect of this herb against OTA-induced increases in serum triglycerides and cholesterol observed in the current investigation is also in agreement with previous reports [79,80]. The protective properties of *Withania somnifera* have been attributed to its biologically active constituents, including alkaloids (anaferine, isopelletierine), saponins, and steroidal lactones (withaferins and withanolides) [39].

The protective effects of *Centella asiatica* on the intestinal tract and vascular integrity have been previously demonstrated [19,20,23]. Similar protective effects against OTA-induced intestinal mucosal damage were observed in rabbits in the present study. Additionally, the previously reported hepatoprotective and nephroprotective properties of *Centella asiatica* [22,23] are in accordance with the findings presented here.

The observed increase in ROS production, ●Asc and ●NO radical concentrations, and -SH conformational changes in OTA-treated rabbits clearly confirms the induction of oxidative stress. Several studies have demonstrated that OTA-contaminated feed leads to enhanced oxidative stress in rabbits. For instance, Prabu et al. [63] reported significant increases in ROS production during intoxication with 1 ppm OTA in rabbits. In the same study, antioxidant enzyme activities such as CAT and SOD, as well as levels of malondialdehyde (MDA), a key biomarker of lipid peroxidation, were significantly elevated in the liver and kidneys on days 30 and 60 compared to controls. In the present study, a similar increase in CAT and MDA was observed; however, SOD activity was decreased, a phenomenon often associated with severe degenerative changes and antioxidant system exhaustion.

In another study, significantly elevated serum levels of MDA and ●NO were reported in rabbits exposed to 0.3 mg/kg OTA [50], while increased MDA levels have also been observed at low OTA doses (19 µg/kg) [57].

The toxicity of OTA is primarily attributed to its inhibitory effects on endogenous and exogenous enzyme systems, leading to increased lipid peroxidation, inhibition of protein synthesis (as reflected by PO and PC markers), and disruption of mitochondrial phosphate transport in the liver and kidneys. In this context, the use of medicinal plants such as *Withania somnifera*, *Centella asiatica*, and *Silybum marianum*, possessing strong antioxidant properties, represents a promising strategy for mitigating OTA-induced toxicity. These bioactive compounds may effectively attenuate oxidative and nitrosative stress and help restore redox homeostasis in rabbits [19,42].

*Centella asiatica* has also been reported to enhance growth performance, immune function, and antibacterial defence [22,23], which may contribute to alleviating OTA-induced immunosuppression and preventing secondary oxidative damage. The organoprotective effects of *Silybum marianum* (particularly its commercial extract, silymarin) are associated with reduced MDA levels and decreased ROS production, thereby stabilising cellular membranes and restoring antioxidant defence mechanisms in the liver and kidneys [27,28,29,30,31,34,81,82].

The antioxidant effects of *Withania somnifera* have similarly been linked to its anti-inflammatory, hepatoprotective, and renoprotective properties [39,41,83], primarily through modulation of ROS/RNS (reactive oxygen and nitrogen species) balance and attenuation of oxidative stress, as demonstrated in rabbits [42].

The modulation of inflammation and reduction in oxidative stress (OS) by the three antioxidants (herbal additives) in the present study were also reflected in the less pronounced histological lesions observed in the liver, kidneys, and other internal organs. These herbal additives likely ameliorated OTA-induced oxidative DNA damage, as indicated by the lower increase in 8-OHdG, a marker of oxidative DNA lesions. The reduced intensity of structural damage in lipids and –SH conformational changes in proteins in rabbits protected by herbal additives may result from a series of electron-donating processes that directly convert O2•− radicals and H_2_O_2_ into less reactive species and water, while also regulating Nrf2 (nuclear factor erythroid 2-related factor 2) activity. Oxidative stress induced by ROS/RNS activates Nrf2, which subsequently stimulates HO-1 production. Furthermore, the herbal additives may modulate SOD/CAT-mediated Fenton synthesis, limit the formation of highly reactive ●OH radicals, and attenuate OTA-induced oxidative damage through regulation of the NF-κB pathway, cytokine levels, and cellular GSH-GPx balance [84], as glutathione (GSH) depletion and GPx deficiency represent critical states compromising antioxidant defence mechanisms [15,16,31,40,43].

The three antioxidants appear to enhance endogenous antioxidant enzyme activity (SOD, CAT, GPx-1) and contribute to stable redox modulation of lipid peroxidation (via regulation of ROS, ●NO, and MDA), cytokine responses, and OS-related disturbances following OTA exposure. In early stages of oxidative stress, a compensatory cellular response is typically observed, involving upregulation of antioxidant enzymes. Elevated CAT activity reflects an active response to oxidative stress, more pronounced in OTA-treated rabbits and less evident in rabbits supplemented with herbal additives. The increased activities of SOD and GPx-1 in the herb-supplemented groups suggest enhanced endogenous antioxidant defence and reduced oxidative stress compared with rabbits receiving OTA alone.

Notably, supplementation with *Centella asiatica* and *Silybum marianum* in OTA-contaminated feed resulted in significant attenuation of OTA toxicity and marked enhancement of antioxidant defence mechanisms in internal organs and blood. The protective effects observed in the present study are consistent with the well-established antioxidant properties of *Silybum marianum*, *Withania somnifera*, and *Centella asiatica*, which have been attributed to their ability to scavenge reactive oxygen species, activate Nrf2-dependent antioxidant pathways, and suppress oxidative tissue injury [15,31,40], as confirmed in the present study. The protective effects were less pronounced for silymarin and *Withania somnifera*. These findings highlight the protective role of *Centella asiatica* and *Silybum marianum*, followed by silymarin and *Withania somnifera*, against OTA-induced oxidative damage, as evidenced by reduced hepatic, renal and pancreatic lipid peroxidation, together with decreased ROS production and ●NO and ●Asc levels compared with the OTA-treated group.

Interestingly, the administration of *Centella asiatica* and *Silybum marianum* alleviated OTA-induced structural alterations by restoring redox homeostasis. These supplements appear to support tissue remodelling and reduce fibrotic processes by inhibiting collagen deposition (Hyp, *p* < 0.05; COL1A1, *p* < 0.05) and restoring ascorbic acid levels [85]. This effect is probably mediated through attenuation of oxidative stress, which limits ROS-induced activation of profibrotic pathways, reduces collagen synthesis, and facilitates normal extracellular matrix remodelling. The elevated COL1A1 levels observed in kidneys following OTA exposure may explain the increased connective tissue proliferation and collagen deposition, which is consistent with the pathomorphological findings in kidneys of OTA-treated rabbits.

The pronounced degenerative changes observed in the kidneys of OTA-treated rabbits were accompanied by significantly increased KIM-1 levels, consistent with renal tubular injury. KIM-1 is a sensitive biomarker of proximal tubular damage and has previously been reported to be elevated in rabbits with OTA-induced nephrotoxicity [13]. In contrast, rabbits receiving herbal supplementation exhibited significantly lower KIM-1 levels, indicating attenuation of OTA-induced renal injury, in agreement with the histopathological findings.

The increased Hyp concentration in OTA-exposed rabbits is commonly associated with fibrosis induced by chronic oxidative stress [86], and correlates positively with MDA and ●NO levels [87]. This process is accompanied by increased collagen synthesis, as also evidenced by the elevated COL1A1 expression observed in the OTA-treated rabbits. The increased Hyp concentration observed in OTA-exposed rabbits, together with the upregulation of COL1A1, reflects enhanced collagen synthesis and extracellular matrix deposition, which is consistent with the pathomorphological findings in the kidneys of OTA-treated rabbits. Oxidative stress induced by OTA stimulates profibrotic pathways, resulting in excessive collagen accumulation. Because hydroxyproline is a major amino acid of collagen, elevated Hyp concentrations are widely recognised as a biochemical indicator of tissue fibrosis. The positive correlation of Hyp with MDA and ●NO further supports the contribution of oxidative stress to OTA-induced fibrogenesis [86,87].

Elevated levels of protein oxidation markers (PO and PC) in OTA-treated rabbits indicate irreversible oxidative damage to proteins, including –SH group alterations and collagen deposition, leading to loss of protein function [88]. These changes correspond well with the granular degeneration observed histologically in the kidneys and liver, which was less pronounced in rabbits receiving herbal supplementation.

The increase in advanced glycation end products (AEGs), which accumulate during chronic oxidative stress, contributes to vascular damage [89], as evidenced by activation of capillary endothelium observed in the kidneys and liver of OTA-treated rabbits. These alterations were less evident in rabbits supplemented with herbal additives.

In the present study, it was observed that OTA-toxicosis in rabbits can reduce antioxidant enzyme activities (SOD and GPx) and total antioxidant capacity, resulting in increased ROS production and lipid peroxidation (MDA). OTA exposure also triggered inflammatory responses, as indicated by elevated levels of pro-inflammatory cytokines, including TNF-α, IL-6, IL-1β, and IFN-γ [90], which was confirmed in this study. However, all investigated herbal additives demonstrated protective effects against OTA-induced oxidative stress. Additionally, oxidative stress may induce a compensatory increase in SIRT-1 expression as a protective mechanism in response to decreased antioxidant enzyme activity and cellular damage, as also observed in the present study.

## 5. Conclusions

The present study demonstrates that dietary supplementation with *Silybum marianum*, *Centella asiatica*, *Withania somnifera*, and silymarin effectively attenuated OTA-induced toxicity in rabbits by reducing oxidative stress, improving growth performance, preserving the structural integrity of internal organs, and restoring metabolic homeostasis. Among the tested additives, *Silybum marianum* and *Centella asiatica* exhibited the strongest protective effects. These findings suggest that herbal feed additives may represent a practical and safe complementary strategy to conventional mycotoxin binders for reducing the adverse effects of naturally OTA-contaminated feeds in rabbit production. Their application may help rabbit producers utilise feeds containing low levels of OTA more safely, thereby reducing economic losses associated with feed rejection, impaired growth, and health deterioration. Further studies should evaluate combinations of herbal additives with commercial mycotoxin binders under practical field conditions.

## Figures and Tables

**Table 1:** Amount of OTA and putative antidotes in experimental feeds of rabbits.

Group	Added OTA in Feed (ppm—mg/kg)	Added Herbal Additives (Antidotes) in Feed (ppm—mg/kg)
SM + OTA	2	*Silybum marianum fruits* powder—5000 ppm
WS + OTA	2	*Withania somnifera radix* powder—4000 ppm
CA + OTA	2	*Centella asiatica leaves* powder—4600 ppm
SIL + OTA	2	Extract Powder of Silymarin—25,000 ppm
OTA	2	none
CONTROL	none	none

**Table 2:** Mean values of feed intake, feed conversion ratio (FCR) and b.w. (body weight) of rabbits (n = 8 per group) supplemented with various putative antidotes and/or OTA for 30 days.

Group	Feed Intake (kg/per Rabbit)	FCR (kg/kg.b.w.)	b.w. (kg)
SM + OTA	3.54	2.10	1.68 ± 0.06 ^a^
WS + OTA	3.75	2.12	1.77 ± 0.05 ^ab^
CA + OTA	3.54	1.89	1.87 ± 0.05 ^b^
SIL + OTA	3.59	2.05	1.75 ± 0.07 ^a^
OTA	3.45	2.33	1.48 ± 0.06 ^a^
Control	3.76	1.86	2.02 ± 0.04 ^b^

± SEM (standard error of the mean). Means sharing the same superscript are not significantly different from each other (*p* < 0.05). ^a^ Significant difference compared to the CONTROL group (*p* < 0.05). ^b^ Significant difference compared to the OTA group (*p* < 0.05).

**Table 3:** Mean serum values of glucose, albumin, total protein (TP), cholesterol (Chol), creatinine (Creat), and urea (BUN) in groups of rabbits (n = 8, on day 30) given various putative antidotes and/or OTA.

Group	Glucose mmol/L	Albumin g/L	TP g/L	Chol mmol/L	Creat µmol/L	BUN mmol/L
SM + OTA	2.64 ± 0.26 ª	42.00 ± 3.42	55.37 ± 3.89	2.32 ± 0.38	96.07 ± 2.40 ª	8.40 ± 0.65
WS + OTA	2.33 ± 0.06 ª	41.00 ± 2.68	73.50 ± 7.95 ^b^	2.02 ± 0.32	97.63 ± 2.96 ª	7.90 ± 0.30
CA + OTA	2.37 ± 0.12 ª	50.62 ± 3.99 ^b^	72.37 ± 6.70 ^b^	1.86 ± 0.11	91.92 ± 2.34	8.70 ± 0.33 ª
SIL + OTA	2.93 ± 0.14 ª	35.50 ± 1.45	51.50 ± 2.30	1.68 ± 0.28	95.70 ± 5.64 ª	8.63 ± 0.32 ª
OTA	2.94 ± 0.16 ª	35.12 ± 1.95	49.75 ± 2.21	2.53 ± 0.43	105.06 ± 12.08 ª	9.00 ± 0.33 ª
CONTROL	3.92 ± 0.31 ^b^	42.50 ± 2.24	58.87 ± 2.13	1.53 ± 0.11	72.21 ± 4.48 ^b^	7.04 ± 0.26 ^b^

± SEM (standard error of the mean). ^a^ Significant difference compared to the CONTROL group (*p* < 0.05). ^b^ Significant difference compared to the OTA group (*p* < 0.05).

**Table 4:** Mean serum values of aspartate aminotransferase (AST), alanine aminotransferase (ALT), alkaline phosphatase (ALP), and triglycerides (TGs) in groups of rabbits (n = 8) given various putative antidotes and/or OTA.

Group	AST U/L	ALT U/L	ALP U/L	TG mmol/L
SM + OTA	45.00 ± 2.13 ^b^	77.12 ± 2.98 ª	473.50 ± 22.57	1.00 ± 0.05 ^b^
WS + OTA	45.87 ± 2.32 ^b^	72.62 ± 2.92 ª	545.88 ± 17.19 ª	1.05 ± 0.03
CA + OTA	41.62 ± 3.34 ^b^	69.00 ± 2.95 ª	518.75 ± 21.35 ª	1.01 ± 0.04 ^b^
SIL + OTA	60.50 ± 6.43 ª^b^	74.12 ± 4.24 ª	527.75 ± 43.19 ª	1.18 ± 1.11
OTA	92.75 ± 7.70 ª	77.50 ± 3.91 ª	583.50 ± 53.02 ª	1.34 ± 0.11 ª
CONTROL	31.37 ± 2.10 ^b^	53.00 ± 2.68 ^b^	353.38 ± 24.44 ^b^	0.99 ± 0.07 ^b^

± SEM (standard error of the mean). ^a^ Significant difference compared to the CONTROL group (*p* < 0.05). ^b^ Significant difference compared to the OTA group (*p* < 0.05).

**Table 5:** Pathohistological examination in internal organs of rabbits from groups (n = 8) exposed to various putative antidotes and/or OTA on day 30 and day 80 *.

Kind of Pathohistological Lesion	OTA	OTA + CA	OTA + WS	OTA + SM	OTA + SIL
**Pathohistological lesions in kidneys**					
Degenerative changes in proximal tubules	+++++	++	++	++	++
Hyperaemia of peritubular capillaries	++	+	+	+	+
Focal mononuclear infiltration in interstitium	+++	+	+	+	+
Activation of capillary endothelium	++	+	+	+	+
Connective tissue proliferation on day 80 *	+++	-	+	-	+
Hypercellularity or sclerosis of glomeruli on day 80 *	+++	+	+	+	-
Atrophy of tubules and retention cysts on day 80 *	+++	-	+	-	-
**Pathohistological lesions in liver**					
Granular or vacuolar degeneration in hepatocytes	+++++	++	++	++	++
Activation of Kupffer’s cells or capillary endothelium	++	+	+	+	+
Hyperaemia or perivascular mononuclear infiltration	+++	+	+	+	+
Bile duct proliferation & peribiliary fibrosis or oedema	+++	++	++	++	++
**Pathohistological lesions in myocardium**					
Hyperaemia of vessels	++	+	+	+	+
Granular degeneration or lytic changes	++	+	+	+	+
Focal mononuclear infiltration	++	+	-	+	+
**Pathohistological lesions in lung**					
Hyperaemia of vessels and siderocytes	++	+	+	+	+
Peribronchial/perivascular mononuclear infiltration	++	+	+	+	+
Focal pneumonia or proliferation of alveolar epithelium	++	+	+	+	+
**Pathohistological lesions in spleen**					
Degenerative changes in white pulp/lymph follicles	++	+	-	+	-
Depletion of cells and reduction in size or number of lymph follicles/white pulp	+++	++	+	+	-
**Pathohistological lesions in thymus**					
Degenerative changes or depletion of cells in cortex	+++	++	+	+	+
**Pathohistological lesions in intestines**					
Degenerative changes in surface/glandular epithelium	++	+	+	+	+
Hyperaemia of vessels	++	+	+	+	+
**Pathohistological lesions in brain**					
Pyknotic or lytic changes in neurons or brain substance	++	+	+	+	+
Pericapillary and pericellular oedema	++	+	+	+	+
Local microglia proliferation	++	-	-	-	-
Pericapillary lymphocytic infiltration	++	-	-	-	-
**Pathohistological lesions in cerebellum**					
Lytic changes in Purkinje cells and *lamina medullaris*	++	+	+	+	+
**Pathohistological lesions in testicles**					
Degenerative changes in seminiferous tubules and/or interstitial oedema on day 80 *	++	+	+	+	+
**Pathohistological lesions in ovary**					
Proliferation of connective tissue and oedema in interstitium on day 80 *	++	+	+	+	+
Hyperaemia of vessels	+	-	-	-	-

- No damage. + Slight damage or damage observed occasionally in a few rabbits. ++ Slight damage in all rabbits or moderate damage in less than half of the rabbits. +++ Moderate damage in all rabbits and/or severe damage in less than half of the rabbits. +++++ Severe damage in all rabbits. *Damages observed mainly on day 80. CA: *Centella asiatica*/WS: *Withania somnifera*/SM: *Silybum marianum*/SIL: silymarin.

## Data Availability

The original contributions presented in this study are included in the paper. Further inquiries can be directed to the corresponding author.

## Abbreviations

The following abbreviations are used in this manuscript:

OTA	ochratoxin A
ppm	mg/kg
AST	aspartate aminotransferase
ALT	alanine aminotransferase
ALP	alkaline phosphatase
GLU	glucose
TP	total protein
ALB	albumin
CHOL	cholesterol
TG	triglycerides
CR	creatinine
BUN	blood urea nitrogen
OS	oxidative stress
ROS	Reactive Oxygen Species
●NO	Nitric Oxide
●Asc	Ascorbate
PO	“Protein Oxidation” or “SH conformations/albumin”
COL1A1	Procollagen type I alpha 1
MDA	lipid peroxidation (malondialdehyde)
KIM-1	kidney injury molecule-1
Hyp	Hydroxyproline
AEGs	advanced glycation end products
HO-1	heme oxygenase-1
CAT	catalase
8-OHdG	8-hydroxy-2′-deoxyguanosine
SOD	superoxide dismutase
SIRT-1	Sirtuin 1
GPx-1	glutathione peroxidase-1
PC	protein carbonyls
INF-γ	interferon-gamma
TNF–α	tumour necrosis factor alpha
